# Use of Doxycycline to Prevent Sexually Transmitted Infections According to Provider Characteristics

**DOI:** 10.3201/eid3001.231152

**Published:** 2024-01

**Authors:** William S. Pearson, Brian Emerson, Matthew Hogben, Lindley Barbee

**Affiliations:** Centers for Disease Control and Prevention, Atlanta, Georgia, USA

**Keywords:** Sexually transmitted infections, doxycycline, antimicrobial resistance, providers, bacteria, prophylaxis, United States

## Abstract

Use of doxycycline to prevent sexually transmitted infections (STIs) may lead to antimicrobial resistance. We analyzed attitudes toward this practice between US providers who commonly and less commonly treat STIs. Providers who more commonly treat STIs are more likely to prescribe prophylactic doxycycline and believe that benefits outweigh potential for increased antimicrobial resistance.

Reports of bacterial sexually transmitted infections (STIs) (e.g., chlamydia, gonorrhea, and syphilis) in the United States are at the highest level in several decades (*1*). A useful tool for preventing STIs may be prophylactic use of doxycycline taken within 72 hours after a sexual encounter (*2*–*5*). However, concerns about development of antimicrobial resistance (AMR) (e.g., in *Neisseria gonorrhea*, which is listed by the Centers for Disease Control and Prevention as an urgent AMR threat), may affect provider attitudes toward prophylactic use of doxycycline (*6*). To determine differences in the practices and beliefs of providers who work with STI patients (STI providers) and do not work with STI patients (non–STI providers) with regard to prophylactic use of doxycycline for STIs and their concerns about potential AMR consequences, we analyzed survey responses.

We analyzed data from the DocStyles panel survey (https://styles.porternovelli.com/docstyles) conducted by SERMO, a social network platform for physicians (https://www.sermo.com) in conjunction with Porter Novelli during September 9–November 3, 2022. Of 1,755 US healthcare providers who responded (response rate 67.0%), we focused on a sample of 1,504 healthcare providers, including family physicians (457, 30.4%), internists (545, 36.2%), obstetrician/gynecologists (251, 16.7%), and nurse practitioners/physician assistants (251, 16.7%). We excluded 251 pediatricians.

We further stratified analyses by the percentage of the providers’ practice focused on clinical management of STIs. Providers were asked, “What proportion of your visits include screening for, diagnosing, or treating sexually transmitted infections?”; the 5 possible responses were “none,” “some, but less than 10%,” “more than 10% up to 25%,” “more than 25% up to 50%,” or “more than 50%.” The 743 respondents whose practice consisted of <10% STI management were considered non–STI providers, and the 761 others were considered STI providers. We further ascertained provider age, sex, specialty, and number of years in practice.

We asked 4 questions about use and beliefs with regard to doxycycline prophylaxis and antimicrobial resistance (Figure), and the 5 response choices were “strongly disagree,” “somewhat disagree,” “neither agree nor disagree,” “somewhat agree,” or “strongly agree.” We used χ^2^ tests to compare the percentage of respondents who chose “strongly agree,” and “agree” between STI providers and non–STI providers. We further tested those differences by using adjusted logistic regression models controlling for provider age, sex, number of years in practice, and specialty (Table).

**Figure:**
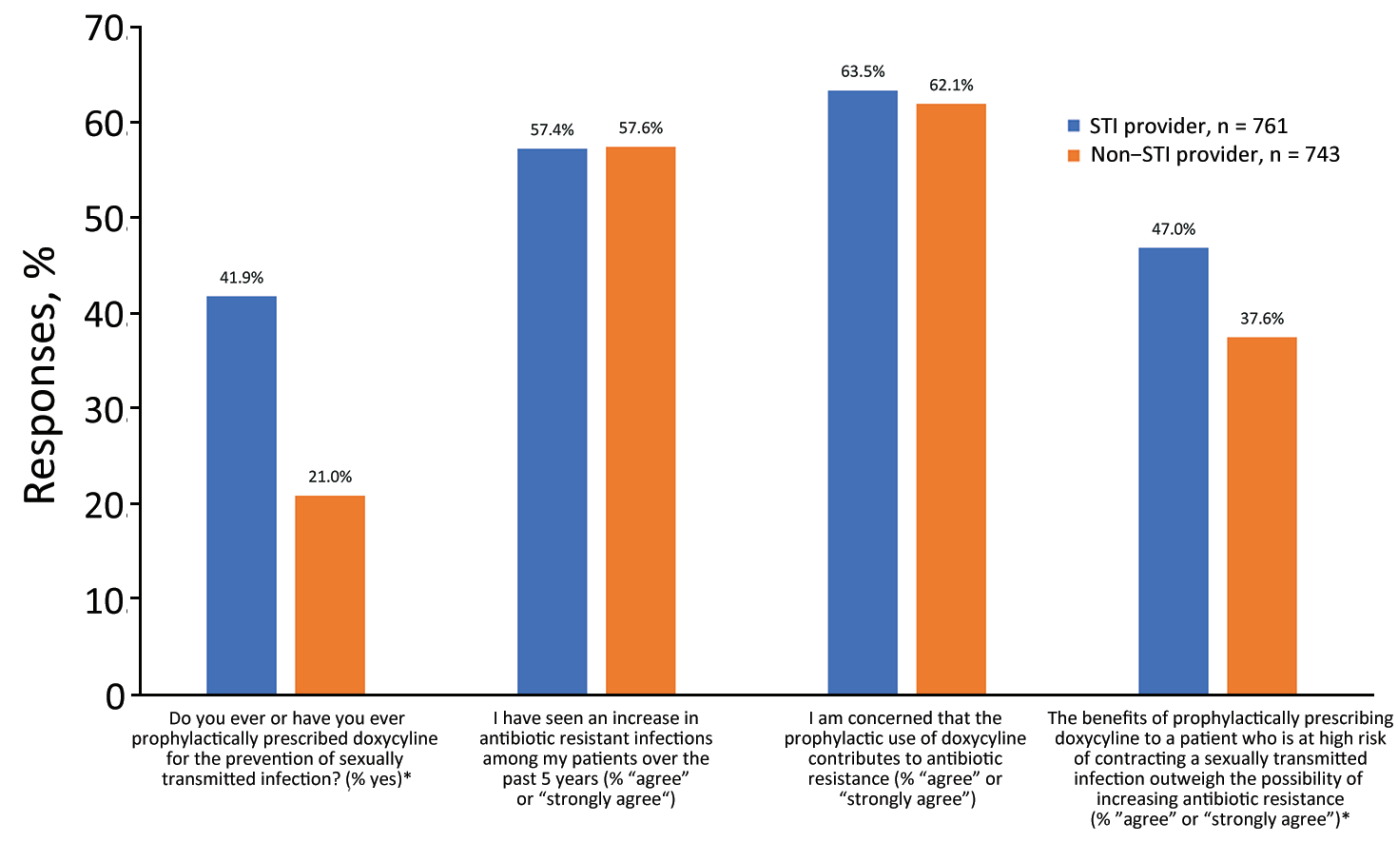
Comparison of prophylactic use of doxycycline and beliefs about antimicrobial resistance among US STI providers and non-STI providers. *Indicates a statistical difference (p<0.05) according to χ^2^ analyses.

**Table:** Logistic regression models comparing STI providers with non–STI providers on practices regarding prophylactic use of doxycycline and beliefs about antimicrobial resistance, United States*

Question/response	Likelihood
Do you ever or have you ever prophylactically prescribed doxycycline for the prevention of a sexually transmitted infection? By prophylactic use, we mean taking doxycycline to prevent infection ahead of or immediately after exposure risk; response: “yes”	
STI provider	aOR 2.76, 95% CI 2.20–3.48
Non–STI provider	Referent
I have seen an increase in antibiotic resistant infections among my patients over the past 5 years; response “agree” or “strongly agree”	
STI provider	aOR 1.00, 95% CI 0.81–1.23
Non–STI provider	Referent
I am concerned that the prophylactic use of doxycycline contributes to antibiotic resistance; response: “agree” or “strongly agree”	
STI provider	aOR 1.09, 95% CI 0.88–1.35
Non–STI provider	Referent
The benefits of prophylactically prescribing doxycycline to a patient who is at high risk of contracting a sexually transmitted infection outweigh the possibility of increasing antibiotic resistance; response: “agree” or “strongly agree”	
STI provider	aOR 1.53, 95% CI 1.25–1.89
Non–STI provider	Referent

*STI providers, n = 761; non–STI providers, n = 743. Controlled for age of provider, sex of provider, specialty of provider, and number of years in practice. aOR, adjusted odds ratio; STI, sexually transmitted infection.

Among STI providers, 41.9% said that they had ever prescribed doxycycline for STI prophylaxis, compared with 21.0% non–STI providers (p<0.01). Among STI providers, 57.4% either strongly agreed or agreed with the statement, “I have seen an increase in antibiotic resistant infections among my patients over the past 5 years,” compared with 57.6% of non–STI providers (p = 0.94). Among STI providers, 63.5% either strongly agreed or agreed with the statement, “I am concerned that the prophylactic use of doxycycline contributes to antibiotic resistance,” compared with 62.1% of non–STI providers (p = 0.57). Among STI providers, 47.0% either strongly agreed or agreed with the statement, “The benefits of prophylactically prescribing doxycycline to a patient who is at high risk of contracting a sexually transmitted infection outweigh the possibility of increasing antibiotic resistance,” compared with 36.6% of non–STI providers (p<0.01) (Figure).

When we used adjusted logistic regression models to control for provider age, sex, specialty, and number of years in practice, STI providers were >2.5 times more likely to have used doxycycline prophylactically for STI prevention (adjusted odds ratio [aOR] 2.76, 95% CI 2.20–3.48) compared with non–STI providers. STI providers were no more likely than non–STI providers to agree that they had seen an increase in AMR among their patients over the past 5 years (aOR 1.00, 95% CI 0.81–1.23) or that the prophylactic use of doxycycline contributes to AMR (aOR 1.09, 95% CI 0.88–1.35). STI providers had ≈50% greater odds than non-STI providers to agree that the benefits of prophylactically prescribing doxycycline for a patient who is at high risk of contracting an STI outweigh the possibility of increasing AMR (aOR 1.54, 95% CI 1.24–1.89).

Our findings suggest that providers whose practice includes >10% STI care are more likely to use doxycycline prophylactically for STI prevention and to believe that the benefits of doxycycline as STI postexposure prophylaxis outweigh the potential for increased AMR compared with providers who do not routinely care for patients with STIs. However, similar proportions of both groups reported concern about the role of prophylactic doxycycline in increasing AMR. Data on the effects that prophylactic use of doxycycline may have on development of AMR are limited (*5*), although antimicrobial use can contribute to the development of AMR (*7*). Additional education on this topic for providers who routinely treat STIs and for providers who routinely prescribe doxycycline will help minimize any potential AMR threats.
